# *In vivo* images of the epidural space with two- and three-dimensional optical coherence tomography in a porcine model

**DOI:** 10.1371/journal.pone.0172149

**Published:** 2017-02-14

**Authors:** Wen-Chuan Kuo, Meng-Chun Kao, Mei-Yung Tsou, Chien-Kun Ting

**Affiliations:** 1 Institute of Biophotonics, National Yang-Ming University, Taipei, Taiwan; 2 Biophotonics and Molecular Imaging Research Center, National Yang-Ming University, Taipei, Taiwan; 3 Institute of Biophotonics, National Yang-Ming University, Taipei, Taiwan; 4 Department of Anesthesiology, Taipei Veterans General Hospital and School of Medicine, National Yang-Ming University, Taipei, Taiwan; Justus Liebig Universitat Giessen, GERMANY

## Abstract

**Background:**

No reports exist concerning *in vivo* optical coherence tomography visualization of the epidural space and the blood patch process in the epidural space. In this study, we produced real-time two-dimensional and reconstructed three-dimensional images of the epidural space by using optical coherence tomography in a porcine model. We also aimed to produce three-dimensional optical coherence tomography images of the dura puncture and blood patch process.

**Methods:**

Two-dimensional and three-dimensional optical coherence tomography images were obtained using a swept source optical coherence tomography (SSOCT) system. Four laboratory pigs were intubated and ventilated after the induction of general anesthesia. An 18-gauge Tuohy needle was used as a tunnel for the optical coherence tomography probe to the epidural space. Two-dimensional and three-dimensional reconstruction optical coherence tomography images of the epidural space were acquired in four stages.

**Results:**

In stage 1, real-time two-dimensional and reconstructed three-dimensional optical coherence tomography of the lumbar and thoracic epidural space were successfully acquired. In stage 2, the epidural catheter in the epidural space was successfully traced in the 3D optical coherence tomography images. In stage 3, water injection and lumbar puncture were successfully monitored in all study animals. In stage 4, 10 mL of fresh blood was injected into the epidural space and two-dimensional and three-dimensional optical coherence tomography images were successfully acquired.

**Conclusions:**

These animal experiments suggest the potential capability of using an optical coherence tomography-based imaging needle in the directed two-dimensional and three-dimensional visualization of the epidural space. More investigations involving humans are required before optical coherence tomography can be recommended for routine use. However, three-dimensional optical coherence tomography may provide a novel, minimally invasive, and safe way to observe the spinal epidural space, epidural catheter, lumbar puncture hole, and blood patch.

## Introduction

Despite continual advancements in medical imaging technology, several problems make noninvasive direct visualization of the spinal epidural space (ES) without radiation exposure difficult to attain. Complex bony structures with small access spaces limit the application of surface ultrasonography, although many studies have proposed solutions and various approaches have been evaluated to resolve these issues. [1,2]. There is a high demand for a fast bedside, intraoperative, real-time imaging modality for use in clinical anesthesia and in the injection of blood patches for postdural puncture headache and so on.

Optical coherence tomography (OCT) [3], which does not involve any radiation, can provide high-quality real-time images with a high discriminative ability (15 μm or better). With OCT, it is possible to visualize the surface morphology and underlying tissue microstructures [4,5]. Optical coherence tomography is clinically effective in many fields and in clinical scenarios such as heart imaging for coronary stent insertion [6,7] and retinal examination. In our previous study, we examined the feasibility of using OCT to evaluate the ES and demonstrated that this method had very high sensitivity and specificity [8]. Other authors have also used OCT for detailed anatomical imaging [8–10]. However, no report exists concerning real-time *in vivo* OCT visualization of the ES. In addition, no study has used OCT to demonstrate the blood patch process in the ES.

In this preliminary experimental series conducted on experimental piglets, we aimed to produce intraoperative real-time two-dimensional (2D) and reconstructed three-dimensional (3D) OCT images of the ES. We concurrently aimed to produce 3D OCT images of the dural puncture and blood patch procedure.

## Materials and methods

### Study design

This study was approved by the Institutional Animal Care and Use Committee of Taipei Veterans General Hospital (Taipei, Taiwan). We conducted all four stages of our optical study on an anesthetized porcine model. All surgery was performed under Isoflurane anesthesia, and all efforts were made to minimize suffering.

### The OCT probe and system

**Fig 1:**
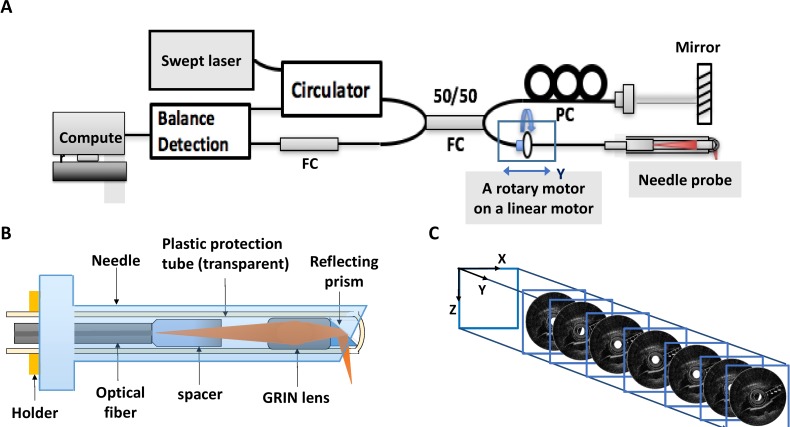
Schematic images of the optical coherence tomography probe and system. (A) The optical setup of the swept source optical coherence tomography (SSOCT) system with the needle probe. (B) The enlarged view of the optical coherence tomography (OCT) probe design. (C) Two-dimensional (2D) OCT imaging is performed by circularly scanning the optical probe within the puncture needle using a rotary motor. Adjacent frames from each rotation are stacked to generate the 3D volume.

Two-dimensional and 3D OCT imaging was acquired using a swept source OCT (SSOCT) setup. The design of our fiber needle SSOCT imaging system has been described in detail [8]. In brief, an SSOCT system is used with a needle probe (Fig 1A). The enlarged figure of the needle probe in Fig 1B shows the single gradient index lens configuration combined with an optical fiber, spacer, and a reflecting prism (i.e., BK7 aluminum-coated prism). This optical probe was constructed to guide and focus light on the tissues. The probe was then connected to a rotary motor, covered by a plastic catheter (with a 0.9-mm outer diameter), and placed into an 18-gauge puncture needle (with a 1.2-mm inner diameter and 1.6-mm outer diameter). The “holder” shown in Fig 1B was designed to keep the probe from moving in and out of the needle during the rotation. The 2D OCT images were acquired by circumferentially scanning the optical probe with a rotational motor, and the rotation of the optical probe was translated by a linear motor in the Y direction so that adjacent frames from each rotation could be stacked to generate a 3D volume (Fig 1C).

In the OCT system, which has high axial and lateral resolutions, the image structure is clear and the image shows closer representation of the actual tissue slice. The axial resolution of the imaging spot size is governed by the light source in our SSOCT system. The system incorporates a 16-kHz frequency swept laser, which has a 1310-nm center wavelength and 3-dB spectral bandwidth (i.e., larger than 100 nm). The SSOCT system accordingly has an axial resolution of approximately 17.5 μm in air (corresponding to 15 μm in tissue), as calculated and measured in our previously published paper [8].

The lateral resolution of approximately 16 μm in air was determined by the spot size of the focusing light, which was related to the design of the gradient index lens used in the needle probe in our SSOCT system. An enlarged figure of the needle probe is shown in Fig 1B. A matrix formulation of Gaussian optics can be used to obtain analytic calculation for the spot size of the probe. [11]

### Porcine model

A porcine model was used because its spinal anatomy is very close to that of humans [12]. Four laboratory pigs with an average weight of 25 kg were intubated and ventilated, after the induction of general anesthesia with intramuscular tiletamine-zolazepam (5 mg/kg). Isoflurane was then used to maintain anesthesia for the duration of the study. The piglets were placed in the left lateral position for epidural placement. A 18-gauge insulated Tuohy needle (Arrow, Teleflex Incorporated, Morrisville, NC, USA) was used as the tunnel for the OCT probe to reach the ES. The animals were euthanized after the procedure.

### Four experimental conditions

The four experimental conditions used in this study were as follows: in stage 1, real-time 2D and reconstructed 3D OCT of lumbar and thoracic ES were successful obtained; in stage 2, epidural catheter in the ES was traced successfully by the 3D OCT images; in stage 3, the water injection and lumbar puncture were also successful monitored in all the study animals; and in stage 4, 10 ml of fresh blood was

#### Stage 1: Two-dimensional and 3D reconstructed OCT images

The goal of stage 1 of the study was to acquire 2D and 3D reconstructed OCT images of the ES. In the preprocedure, the needle probe was inserted into the ES in the lumbar and thoracic regions, using the loss-of-resistance technique and the paramedian approach. We then put the OCT catheter into the puncture needle. As the tip of the OCT catheter reached the ES, “side-looking” images around the needle tip were acquired. Thus, we opened the “holder” and pushed the fiber probe beyond the needle tip by approximately 10 cm into the space cephalically (i.e., Y direction). A series of 2D circumferential OCT images was continuously built up by rotating the optical probe with a rotary motor within the ES channel, as the OCT probe was pulled back by using a linear motor in the negative Y direction. Adjacent frames from each rotation were stacked to generate the 3D volume.

To achieve a working image, including all signal processing, 0.1 second was required, which was only limited by the scanning frequency of the laser. Thus, a series of 2D images was built up within the ES at a rate of 10 frames per second by continuously rotating the optical probe with a rotary motor at a speed of 10 revolutions per second. Each frame consisted of 790 A-lines with an imaging depth of approximately 2 mm in water. For 3D imaging acquired by using a linear scan distance of 3 cm on a sample and a frame-by-frame distance of 50 μm, one reconstructed 3D volume would include 600 frames and take approximately 60 seconds to acquire. Needle probe placement was also confirmed by ultrasonography (Vivid e, GE Healthcare, London, UK) and radiography with 5.0 mL of contrast medium (i.e., ioxitalamic acid).

#### Stage 2: Epidural catheter in the ES

**Fig 2:**
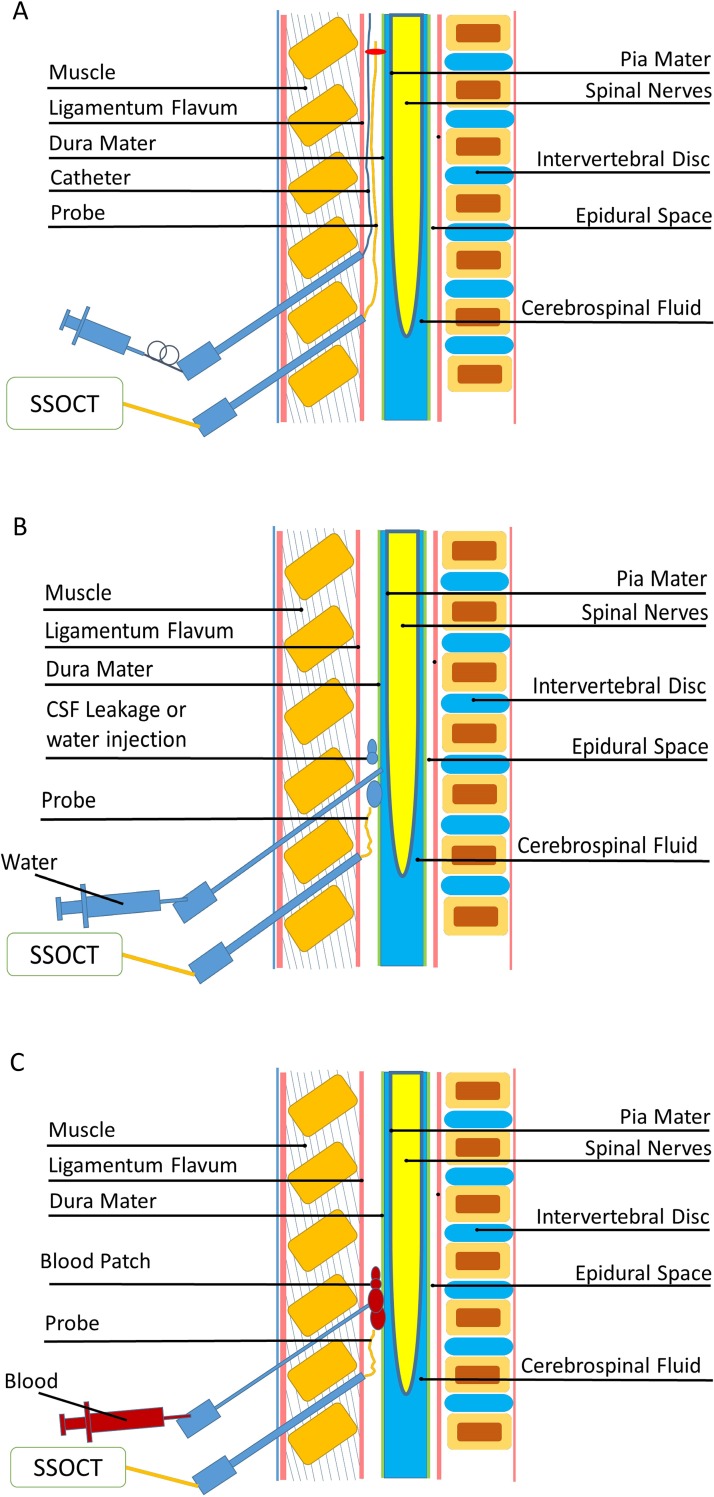
Schematic images of the two-needle method. (A) The two-needle method is used to visualize the epidural catheter in the epidural space (ES) with the OCT probe in real time. (B) In the two-needle method, water is injected into the ES, a lumbar puncture hole is created, and the epidural catheter is observed in real time with the OCT probe. (C) In the two-needle method, the blood patch in the ES is observed with the OCT probe.

In stage 2 of the study, we attempted to demonstrate the ability of OCT to trace the epidural catheter in the ES. We acquired 2D and 3D reconstructed OCT images of the epidural catheter in the ES. Fig 2A shows the two-needle method for observing the catheter in the ES with the OCT probe in real time.

#### Stage 3: Dural puncture and water injection

In stage 3 of the study, a lumbar puncture hole in the dura mater was created, 2D and 3D reconstructed OCT images of cerebrospinal fluid (CSF) leakage in the ES were acquired, and the puncture hole in the dura mater was identified. In addition to the dural puncture, we injected 10 mL of normal saline to observe OCT images of water in the ES. Fig 2B shows the two-needle method used to inject water into the ES, to create a lumbar puncture hole and to observe the catheter in real time with the OCT probe.

#### Stage 4: Blood patch

After the lumbar puncture, there was a hole in the dura mater of the experimental piglet. In stage 4 of this study, 10 mL of fresh blood was withdrawn from a vein and then injected into the ES adjacent to the punctured site, so that the effect of blood patching could be observed. We acquired 2D and 3D reconstructed OCT images of the blood patch 5 min and 60 min after fresh blood was injected into the ES. Fig 2C shows the method of OCT image observation.

## Results

**Fig 3:**
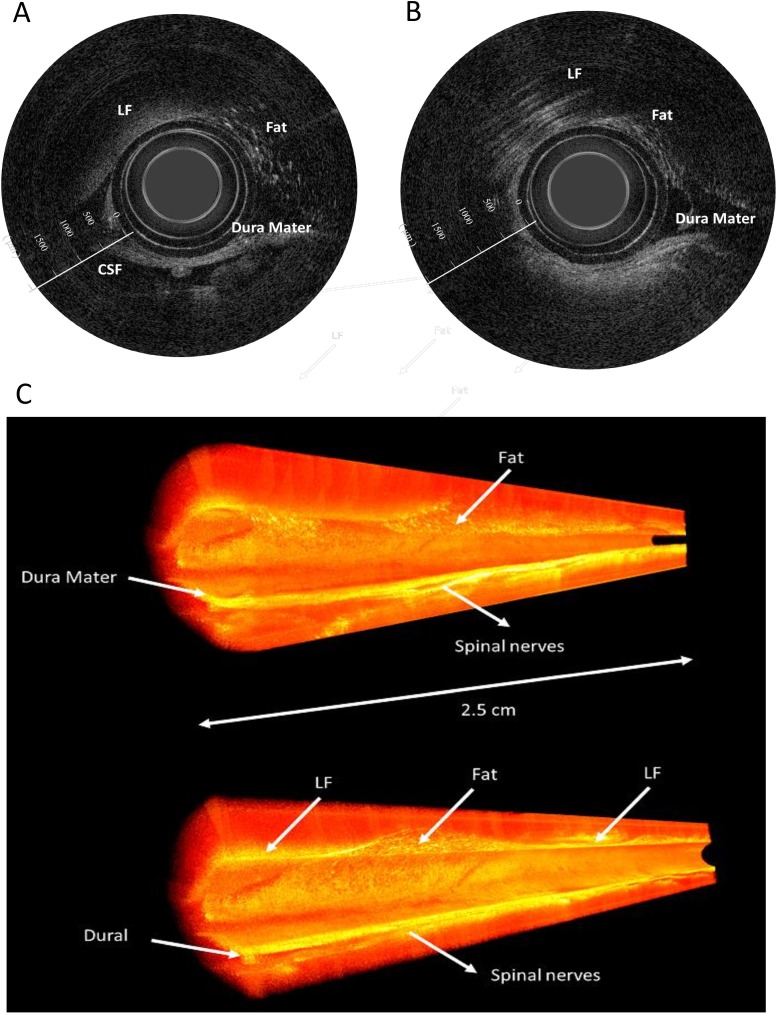
Visualization of the epidural space using optical coherence tomography. (A) (B) Two-dimensional (2D) optical coherence tomography (OCT) images of the epidural space. (C)Three-dimensional (3D) reconstructed OCT images in the XYZ axis format.CSF, cerebrospinal fluid; LF, ligamentum flavum.

In stage 1, real-time 2D and reconstructed 3D OCT images of the lumbar and thoracic ES were successfully obtained in all 24 attempts conducted on the experimental piglets. Fig 3A illustrates the typical 2D images of the ES of a piglet. The two signal-rich bands, indicated by white arrows in the center part of the 2D OCT images, occurred because of backscattering from the plastic protection tube. The ligamentum flavum had strong homogeneous signal distributions. The CSF had no backscattering signal. The posterior dura mater appeared as a signal-rich layer. Adipose fat tissue in the ES was easily differentiated by its lattice-like structure. Below the dura mater, multiple nerve roots (indicated by white arrows) appeared as distinctive and homogenous round-to-oval structures. The 3D reconstructed volume is shown in Fig 3B. The signals from the protection tube were deleted for a clearer view of the inner structures inside the ES.

**Fig 4:**
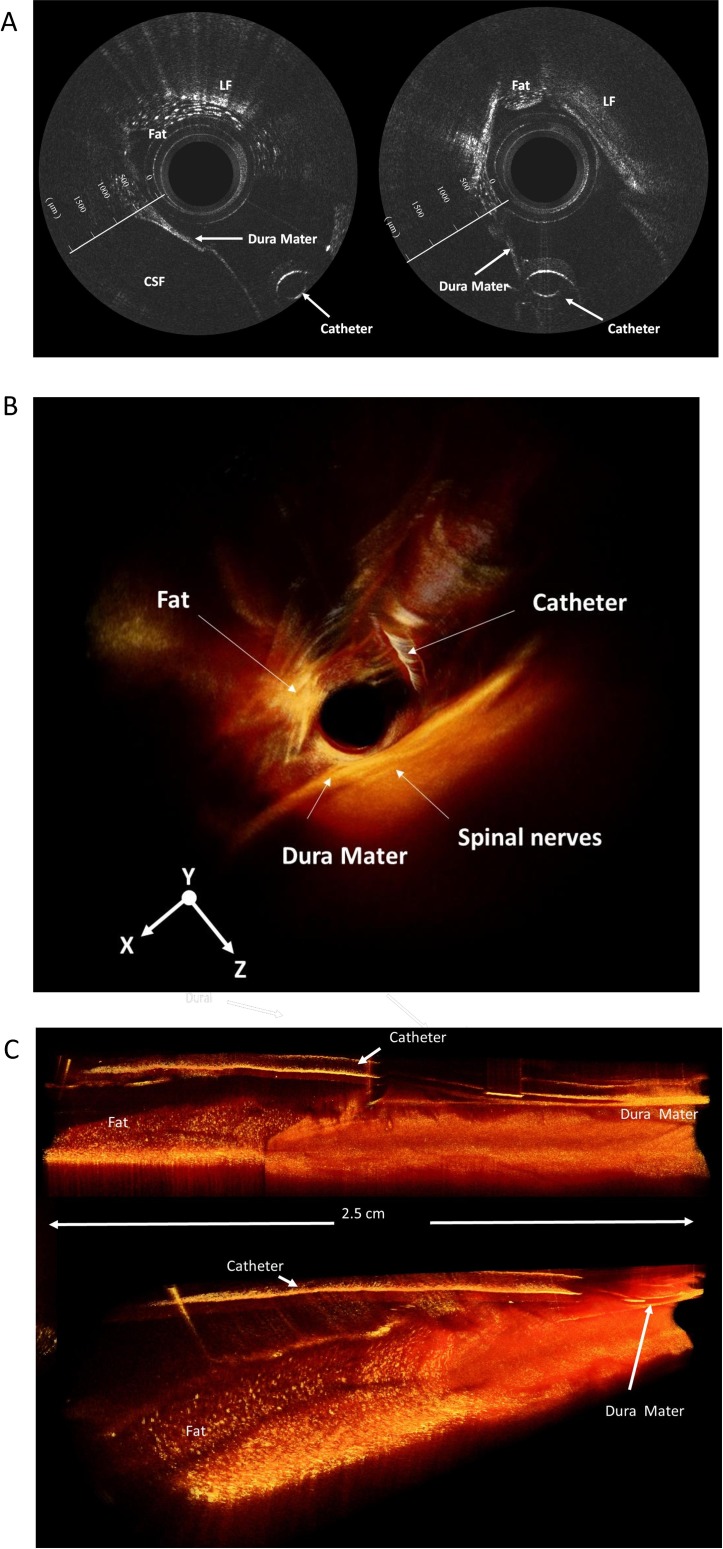
The epidural catheter in the epidural space. The epidural catheter in the epidural space is successfully traced (A) in the 2D image and (B and C) in the 3D OCT images. LF, ligamentum flavum.

In stage 2, the epidural catheter in the ES appears as a hole in the 2D section view (Fig 4A). The catheter and adipose tissue were configured successfully using the reconstructed 3D OCT images in a special angle (Fig 4B). The catheter could be reliably observed using 3D OCT in the lumbar and thoracic regions of the piglets in which adipose tissue is randomly distributed in the ES to form epidural fat. (Fig 4C)

**Fig 5:**
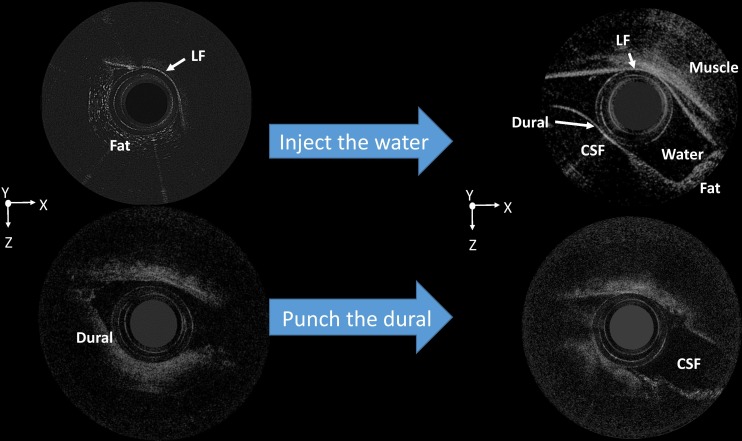
Two-dimensional (2D) optical coherence tomography (OCT) images after water injection and the dura puncture. The injected water and cerebrospinal fluid are visible. CSF, cerebrospinal fluid; LF, ligamentum flavum.

In stage 3, water injection and lumbar puncture were were done successfully in all study animals. Fig 5 shows 2D OCT images of the injected water inside the ES (upper row) and the CSF leakage created by lumbar puncture with an 18-gauge needle (lower row). The ES channel was distended by the water. The same phenomenon was also observed during CSF leakage resulting from the dura puncture.

**Fig 6:**
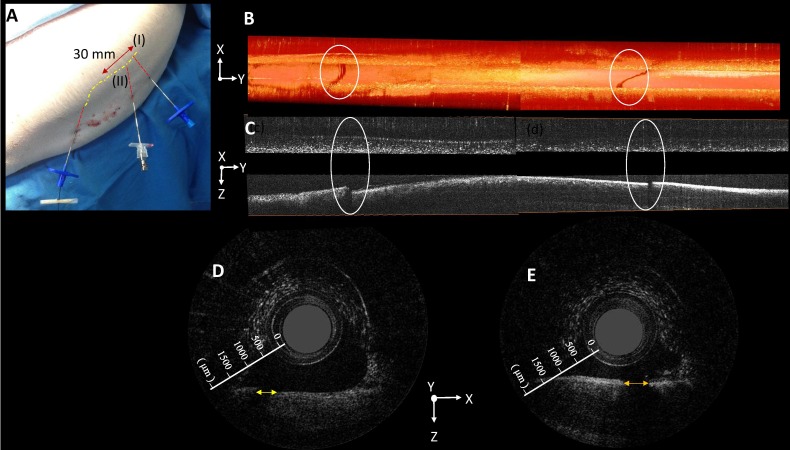
The dural puncture scenario simulated by creating two holes in the dura. (A) For the lumbar puncture, the dura is punctured at two points (i.e., point I and point II) in the back of the piglet. (B) The three-dimensional (3D) reconstructed optical coherence tomography (OCT) image of the puncture holes in the dura mater is shown in the XY plane. (C) The 3D reconstructed OCT image of the puncture holes in the dura mater is shown in the YX plane. (D) and (E) The 2D OCT images reveal that each puncture (point I and point II, respectively) produces discontinuity in the highly scattering dura mater layer, as shown in the XZ plane.

We simulated the dural puncture effect by creating two holes [i.e., point (I) and point (II)] on the dura (Fig 6A). The red line in Fig 6A indicates the 25-mm scanning distance in the 2D/3D OCT images within the two lumbar puncture holes in the dura mater. The frame-by-frame distance was 15 μm. Each puncture produced a fissure in the highly scattering dura mater layer, when visualized using reconstructed 3D OCT, and in the XY plane (Fig 6B). When observed in the YZ plane (Fig 6C) or the XZ plane (Fig 6D and 6E), the effect on the dura appears as a significant conical defect or as a discontinuity subsequent to the puncture by the epidural needle. Approximately 113 frames showed discontinuity in the left punctured hole (Fig 6B). Thus, the lateral size of the dura mater defect was estimated as 15 μm × 113 frames– 1.7 mm. The same calculation showed the lateral size of the right punctured hole was approximately 1.4 mm (Fig 6B). The average size of 1.55 mm was comparable to the size of the needle tip (1.47 mm). No similar defect was observed in the intact (i.e., nonpunctured) dura mater.

**Fig 7:**
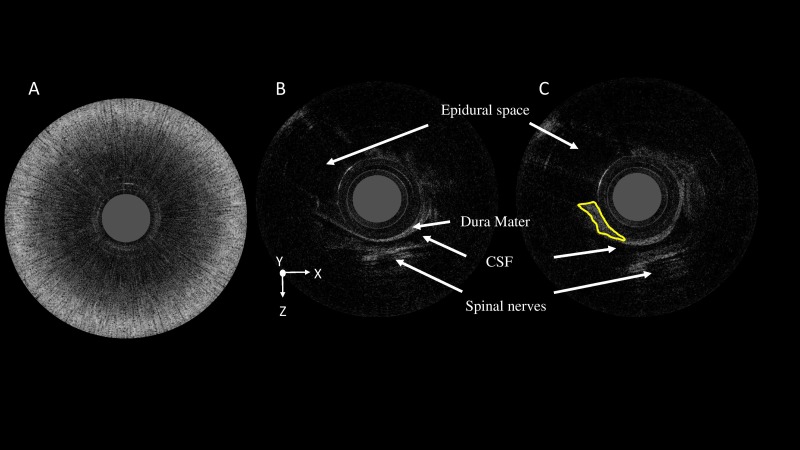
The optical coherence tomography image after the injection of 10 mL of fresh blood into the epidural space. (A) A dark central region with a high level of circular scattering in the two-dimensional (2D) optical coherence tomography (OCT) image when the fiber probe is filled with blood. (B) The normal dura mater signal in the epidural space. (C) The dura mater covered by a blood clot (yellow line) after the blood patch procedure. CSF, cerebrospinal fluid.

In stage 4, 10 mL of fresh blood was injected into the ES, and 2D and 3D OCT images were acquired successfully. Fig 7A shows a dark central region with a high level of circular scattering in the 2D OCT image when the fiber probe was filled with blood. Blood is highly reflected; therefore, the OCT image shows a strong scattering signal. Fig 7B shows the normal dura mater signal in the ES, whereas Fig 7C shows the dura covered by the blood clot after the blood patch procedure. The yellow line encircles the blood clot in Fig 7C, which forms an intense reflected signal, and the thickened dura mater surface.

## Discussion

In this study, our measurements first showed that it was possible to trace the epidural catheter in the ES using 3D OCT, Using OCT, we successfully detected the puncture hole in the dura mater and monitored ES expansion due to CSF leakage subsequent to the dura puncture. The OCT image could be used to target an epidural blood patch (EBP) and check hematoma formation within the space just after the procedure. We have recently shown that the ES can be identified from external tissues, based on the visual details of subsurface morphological information on 2D OCT images [8]. This study extended the 2D images to 3D visualization. More complete results are needed; however, this study acquired *in vivo* 3D scans of the ES. To our knowledge, this is the first such demonstration.

In 1991, epiduroscopy with a flexible optical fiber was introduced into clinical practice [13]. However, its large bore and difficulty of use limit its application in daily epidural block practice and not all pain clinicians and neurosurgeons are familiar with using it. Three-dimensional OCT images provide a novel way to view the ES with a common epidural needle and make it possible for most anesthesiologists to use 3D OCT at the bedside in the operating room and on the ward. Epidural fat, venous plexus, dura mater, and ligamentum flavum are easily identified on 3D OCT images.

Epidural catheters are flexible to avoid vascular and dural puncture. This factor makes the catheter prone to coiling [14], knotting [15,16], kinking[17], and unilateral movement [14] in the ES, especially when it is inserted more than 4–8 cm. An accurate catheter tip position is important for drug delivery and spreading epidural anesthesia. Fluoroscopy [18], motor and sensory-stimulating catheters [19], and near-infrared light [20] have been used to predict clinical catheter function. However, these types of equipment are not applied routinely in epidural anesthesia and have some safety concerns, especially in obstetric applications. Our technique using 3D OCT imaging of the ES has the advantages of detecting the catheter tip and showing the entire route travelled by the catheter in the ES. Tracing the epidural catheter using our OCT image system could avoid several problems associated with epidural catheters.

An autologous EBP has been used to treat headache resulting from intracranial hypotension, which may be induced spontaneously by spinal pathology, surgery, and incidental dura puncture. In general, patches have positive outcomes and an overall efficacy of approximately 90% [21]. However, several complications such as hearing changes [22], vision loss [23], and transient bladder and fecal incontinence [24] have been reported with the procedures. Some trials have suggested the greater efficacy of radiologic-targeted EBPs, compared to blind EBPs, for treating spontaneous intracranial hypotension [21, 25]. Our 3D OCT imaging system may be used to develop a technique for nonradiation-targeted EBP, which may reduce the incidence of complications of blind EBPs and has no radiation exposure.

Traditional radiation-based targeting methods for EBPs include computed tomography (CT) [26], dynamic CT myelography [27], magnetic resonance imaging (MRI) [28, 29], and fluoroscopy [30]. For these radiation-targeted methods, patients need to go to a special therapeutic room with radiation equipment attended by a radiologist or other specialist. Our 3D OCT images could identify the puncture hole in the dura mater, and thereby make it possible for a single anesthesiologist to perform procedures from diagnosis to an EBP at the bedside without additional support and without any radiation exposure. The imaging technique would greatly reduce the waiting time and procedure time for patients, and reduce the duration of a hospital stay and medical costs. Optical coherence tomography imaging could also be applied to check the condition of blood patches after the procedure and make early rescue possible, whereas most radiation-targeted and blind EBPs are only applied, based on the clinical effect after 24 hours.

This method has some limitations. First, the translation of our results to humans is limited because of the anatomical differences between pigs and humans such as smaller spinal canal in pigs. Second, the current completed prototype combined a rotated side-looking fiber probe with a low frequency swept OCT system (–8 kHz A-scan); the image acquisition rate was thus limited to 10 frames per second, which is very low and is sensitive to slow motions such as a patient’s breathing. Because of the recent development of mega-Hertz range swept laser sources, which make the video-rate 3D OCT possible, this capability can significantly impact image-guided applications. Third, an objective judgement tool that can help physicians interpret the OCT images is needed, which we are developing. In our previously published paper [8], we found that OCT images with high sensitivity and specificity could be evaluated by using the characteristic morphological features of different tissues (Table 1 in Ref [8]); however, although high interexpert variation makes this decision greatly divergent.

In conclusion, these preliminary animal experiments suggest the potential capability of an OCT-based imaging needle system for the direct 2D and 3D visualization of the ES. More investigations involving humans are required before OCT can be recommended for routine use. However, 3D OCT may provide a new minimally invasive and safe way to the observe the spinal ES, epidural catheter, lumbar puncture hole, and blood patches. It also may become a safer and faster minimally invasive targeted procedure for blood patches and adhesiolysis guidance.
